# Impact of a countywide smoke-free workplace law on emergency department visits for respiratory diseases: a retrospective cohort study

**DOI:** 10.1186/1471-2466-15-6

**Published:** 2015-01-22

**Authors:** Ivana T Croghan, Jon O Ebbert, J Taylor Hays, Darrell R Schroeder, Alanna M Chamberlain, Véronique L Roger, Richard D Hurt

**Affiliations:** Nicotine Research Program, Department of Internal Medicine, Mayo Clinic, 200 First Street SW, Rochester, MN 55905 USA; Clinical Research Office, Department of Internal Medicine, Mayo Clinic, Rochester, MN USA; Robert D. and Patricia E. Kern Center for the Science of Health Care Delivery, Mayo Clinic, Rochester, MN USA; Division of Biomedical Statistics and Informatics, Department of Health Sciences Research, Mayo Clinic, Rochester, MN USA; Division of Epidemiology, Department of Health Sciences Research, Mayo Clinic, Rochester, MN USA; Division of Cardiovascular Diseases, Department of Internal Medicine, Mayo Clinic, Rochester, MN USA

**Keywords:** Asthma, COPD, Emergency department visits, Secondhand smoke, Smoke-free policies

## Abstract

**Background:**

With the goal of reducing exposure to secondhand smoke, the state of Minnesota (MN), enacted a smoke-free law (i.e., Freedom to Breathe Act) in all workplaces, restaurants, and bars in 2007. This retrospective cohort study analyzes emergency department (ED) visits in Olmsted County, MN, for chronic obstructive pulmonary disease (COPD) and asthma over a five-year period to assess changes after enactment of the smoke-free law.

**Methods:**

We calculated the rates of ED visits in Olmsted County, MN, with a primary diagnosis of COPD and asthma in the five-year period from January 1, 2005 to December 31, 2009. Analyses were performed using segmented Poisson regression to assess whether ED visit rates declined following enactment of the smoke free law after adjusting for potential underlying temporal trends in ED visit rates during this time period.

**Results:**

Using segmented Poisson regression analyses, a significant reduction was detected in asthma-related ED visits (RR 0.814, p < 0.001) but not for COPD-related ED visits following the enactment of the smoke-free law. The reduction in asthma related ED visits was observed in both adults (RR 0.840, p = 0.015) and children (RR 0.751, p = 0.015).

**Conclusions:**

In Olmsted County, MN, asthma-related ED visits declined significantly after enactment of a smoke-free law. These results add to the body of literature supporting community health benefits of smoke-free policies in public environments and their potential to reduce health care costs.

## Background

Tobacco smoke is one of the most common asthma triggers. Asthma may be caused by smoking, and childhood exposure to secondhand smoke (SHS) increases the risk for asthma exacerbations [1]. Chronic obstructive pulmonary disease (COPD) accounts for 73% of smoking-related conditions among current cigarette smokers and 50% among former smokers [2, 3]. Cigarette smoking is the most important risk factor in the development of COPD, as well as the most important modifiable risk factor in reducing the progression and severity of COPD [4, 5]. Reduction in cigarette smoke exposure can reduce the incidence of new cases and exacerbations of COPD and asthma [1, 5–7].

Protecting the public from exposure to tobacco smoke is a key component of the World Health Organization (WHO) Framework Convention on Tobacco Control [8]. After four decades, worldwide smoke-free policies and laws are still works in progress [9–12]. Partial or comprehensive smoke-free workplace laws are in place in 36 of the 50 United States (US) as well as 92 nations worldwide [13]. Nearly 50% of the US population is covered by comprehensive smoke-free regulations and over 80% are covered by partial smoke-free policies. Smoke-free policies and laws have been associated with reduced exposure to SHS and reductions in smoking prevalence [14–18]. The effects of smoke-free policies and laws among populations are difficult to accurately measure due to the number of potential environmental confounders. Despite this limitation of epidemiologic studies, the association between the smoke-free policies and the reduction of SHS exposure has been unequivocally demonstrated [19]. Studies have shown a 72% drop in environmental measures of SHS within one year of smoke-free workplace law implementation and a median decrease of 6% in self-reported exposure to SHS [16, 17]. In 2009, there were approximately 2.1 million asthma-related emergency department (ED) visits in the US, which translated to 69.7 asthma-related visits per 10,000 [20]. In 2010 the rate for COPD-related ED visits was 1.5 million, which translated to 72 ED visits per 10,000 [21]. In this study, we evaluated the impact of the implementation of a state-wide clean indoor air law in 2007 (i.e., Freedom to Breathe Act) on the frequency of ED visits for asthma and COPD [12] within Olmsted County, Minnesota (MN).

## Methods

### Study setting

The 2013 population estimate of Olmsted County, located in southeastern MN, was 147,066 (86.5% white, 51.1% female) [22]. Within this county, two providers (Mayo Clinic and Olmsted Medical Center) deliver nearly all medical care and ED services to county residents. The Rochester Epidemiology Project (REP) [23] allows the linkage of medical records and retrieval of diagnoses and procedures from all sources of care in Olmsted County. This provides a unique infrastructure to analyse disease occurrence and outcomes at the population level. Potential cases identified through the REP can then be validated by applying standardized methods appropriate for each disease entity [24].

### Ascertainment of asthma and COPD ED visits

The MN smoke-free law that was passed on May 16, 2007, and enacted on October 1, 2007, included all workplaces (including bars and restaurants) [12]. The data collected for this review included all ED visits in Olmsted County, MN, from January 1, 2005, through December 31, 2009, providing a window of approximately two years before and after the effective date of the smoke-free law.

All ED visits with the primary diagnosis of COPD (*International Classification of Diseases, Ninth Revision* codes 491–492 and 494–496) or asthma (*International Classification of Diseases, Ninth Revision* code 493) were captured. When multiple ED visits occurred for a single person, all visits were counted since our focus was to assess changes in ED utilization over time for a defined geographic population.

### Statistical analysis

In order to account for potential underlying temporal trends in ED visit rates, analyses were performed using segmented Poisson regression [25]. For all Poisson regression analyses, the ED visit counts for each period-, age-, and sex-combination were used as the unit of observation, with the period-, age-, and sex-specific population counts used as offsets. Since many work places transitioned to smoke-free environments between the passage of the law (5/16/2007) and its implementation (10/01/2007), this calendar period was considered a transition period. Given the limited number of monthly counts during this transition period, the visit counts for May 2007 through September 2007 were excluded rather than modeled as a separate segment. The models included terms to assess for a linear trend prior to passage of the smoke-free law, a step-change with the implementation of the law, and the change in trend after implementation. Due to the broad age range of patients with asthma-related ED visits, analyses were performed overall and separately for adults (≥18 years of age) and children (<18 years of age). Analyses were performed using SAS software (SAS version 9.3; SAS Institute Inc., Cary, NC).

All aspects of the study were approved by the Mayo Clinic and Olmsted Medical Center Institutional Review Boards.

## Results

### Patient characteristics

During the 5-year study period (January 1, 2005 through December 31, 2009), a total of 5,293 ED visits occurred with a primary diagnosis of COPD and 5,906 ED visits with a primary diagnosis of asthma (Table 1).

**Table 1:** **Patient characteristics**

		Asthma		
Characteristic	COPD	Overall	Adults	Children
	n = 5293	n = 5906	n = 4375	n = 1531
Age, y, median (25^th^, 75^th^)	75 (64, 82)	37 (17, 57)	47 (33, 65)	6 (3, 11)
Sex				
Female, n (%)	2551 (48.2)	3619 (61.3)	2994 (68.4)	625 (40.8)
Male, n (%)	2742 (51.8)	2287 (38.7)	1381 (31.6)	906 (59.2)

*Abbreviation: COPD* chronic obstructive pulmonary disease.

### ED incidence rates for COPD and asthma

**Figure 1:**
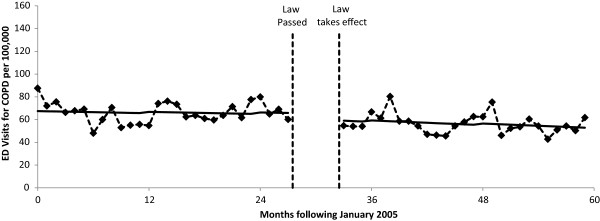
**ED visit rates for COPD before and after smoke-free law.** Visit rates are presented monthly from 1/1/2005 through 12/31/2009. The vertical dashed lines indicate the time period between when the law was passed and when the law took effect. Solid lines represent the predicted rates from segmented Poisson regression.

**Figure 2:**
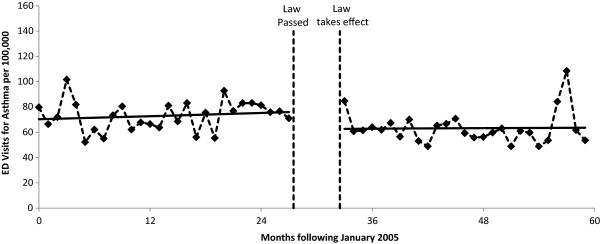
**ED visit rates for asthma before and after smoke-free law.** Visit rates are presented monthly from 1/1/2005 through 12/31/2009. The vertical dashed lines indicate the time period between when the law was passed and when the law took effect. Solid lines represent the predicted rates from segmented Poisson regression.

**Figure 3:**
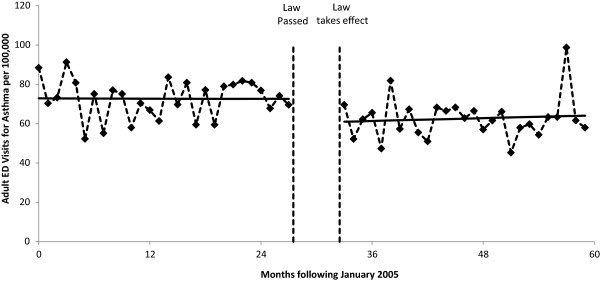
**Adult ED visit rates for asthma before and after smoke-free law.** Visit rates are presented monthly from 1/1/2005 through 12/31/2009. The vertical dashed lines indicate the time period between when the law was passed and when the law took effect. Solid lines represent the predicted rates from segmented Poisson regression.

**Figure 4:**
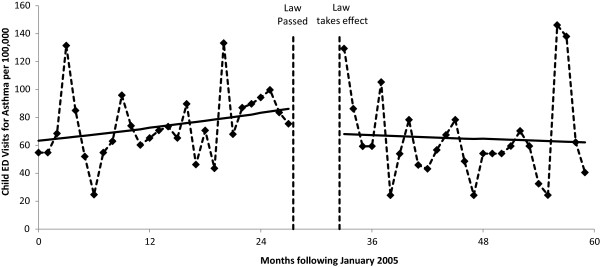
**Child ED visit rates for asthma before and after smoke-free law.** Visit rates are presented monthly from 1/1/2005 through 12/31/2009. The vertical dashed lines indicate the time period between when the law was passed and when the law took effect. Solid lines represent the predicted rates from segmented Poisson regression.

The results of the segmented Poisson regression analyses are summarized in Table 2 and Figures 1, 2, 3 and 4. For COPD, the implementation of the smoke-free law was not found to be associated with a significant step change in ED visit rates (p = 0.158) or with a change in the trend (p = 0.313; Figure 1). There was evidence that the implementation of the smoke-free law was associated with a downward step change in ED visits for asthma (RR = 0.814, 95% CI 0.722 to 0.966; p < 0.001; Figure 2). When the analysis of ED visits for asthma was restricted to adults a similar downward step change was detected (RR = 0.840, 95% CI 0.729 to 0.966; p = 0.015; Figure 3). For children, in addition to a downward step change with the implementation of the smoke-free law (RR = 0.751, 95% CI 0.595 to 0.947; p = 0.015), there was a significant (p = 0.027) change in the trend over time, such that the significant (p = 0.015) increasing trend observed prior to the smoke-free law being passed was no longer present after the law was enacted (p = 0.424; Figure 4).

**Table 2:** **Segmented poisson regression results** ^*^

	Monthly trend before law was passed (β _1_ )	Step change when law took effect (β _2_ )	Change in trend after law took effect (β _3_ )	Monthly trend after law took effect (β _1 +_ β _3_ )
COPD				
β (SE)	-0.002 (0.002)	-0.091 (0.065)	-0.004 (0.004)	-0.006 (0.003)
RR (95% CI)	0.998 (0.993, 1.002)	0.913 (0.804, 1.036)		0.994 (0.989, 0.999)
p value	0.331	0.158	0.313	0.028
Asthma				
Overall				
β (SE)	+0.003 (0.002)	-0.206 (0.061)	-0.002 (0.003)	+0.000 (0.003)
RR (95% CI)	1.003 (0.998, 1.007)	0.814 (0.722, 0.918)		1.000 (0.995, 1.005)
p value	0.236	<0.001	0.502	0.876
Adults				
β (SE)	-0.000 (0.003)	-0.175 (0.072)	+0.002 (0.004)	+0.002 (0.003)
RR (95% CI)	1.000 (0.994, 1.005)	0.840 (0.729, 0.966)		1.002 (0.996, 1.008)
p value	0.922	0.015	0.611	0.552
Children				
β (SE)	+0.011 (0.004)	-0.287 (0.118)	-0.015 (0.007)	-0.004 (0.005)
RR (95% CI)	1.011 (1.002, 1.020)	0.751 (0.595, 0.947)		0.996 (0.986, 1.006)
p value	0.015	0.015	0.027	0.424

*The segmented Poisson regression model adjusted for age and sex provides estimates for the trend in ED visits over time prior to the law being passed (β_1_), the step change after the law took effect (β_2_) and the change in trend after the law took effect (β_3_). The sum of β_1_ and β_3_ corresponds to the trend over time after the law took effect.

*Abbreviation: COPD* chronic obstructive pulmonary disease.

## Discussion

In this study, we observed a reduction in asthma-related ED visits for both adults and children following the enactment of the smoke-free workplace law. Other investigations have evaluated the association between smoke-free laws and asthma incidence, symptoms, hospitalizations, and asthma-related ED visits [14, 15, 26, 27] with similar findings. Our findings are in keeping with a number of studies demonstrating the health benefits of smoke-free indoor air laws, particularly with regard to asthma. Investigators in Kentucky evaluated ED visits for asthma from four different hospitals, comparing rates before and after implementation of a smoke-free law. A 22% reduction in asthma-related ED visits after implementation of the smoke free law was observed [26]. The percentage reduction in asthma-related ED visits is similar to our results. In slight contrast, a study in England evaluating the impact of smoke-free laws on emergency room admissions observed only a 4.9% reduction in asthma-related ED visits after implementation of the smoke-free laws [28]. In a multi-state study, hospital discharge rates for asthma were compared between 12 states with strong smoke-free laws and 5 with weak or no smoke-free laws. A significant reduction in hospital discharges for asthma was observed in states with strong smoke-free laws [14]. Additionally, a recent review of 45 studies focusing on 33 smoke-free laws found a 24% reduction in overall respiratory disease hospitalizations and deaths [29]. This study, with a median follow-up of two years after smoke-free law implementation, observed that the risk of hospitalization and death from tobacco-caused disease does not change with longer follow-up, suggesting that risk reductions will likely be sustained over time [29]. An Arizona study comparing hospital admissions between counties with and without smoke-free policies for the four major tobacco-related diseases (acute myocardial infarction, angina, stroke, and asthma) found a reduction in hospital admissions for these diseases among those counties with a smoke-free policy; notably, asthma admissions were reduced by 22% [30].

When a smoke free policy has been enacted in a stepwise fashion, an association with asthma incidence has not always been found to be consistent between steps. Two studies evaluated more than two periods during smoke-free policy enactment and found little to no association. Investigators in Toronto, Canada who reviewed multiple periods during smoke-free policy enactment and implementation found a large decrease in respiratory conditions overall when the smoke-free law was implemented for restaurant settings, but found no subsequent change when the law was extended to other indoor settings [31]. A study conducted in Geneva looked at hospital admissions in one hospital during four differing time periods in the smoke-free law enactment and implementation process; and although a similar analysis (adjusted Poisson Regression) was used, they found the reverse of our findings. In their study, they found a reduction in COPD hospital admissions but not in asthma admissions [32].

Data concerning the impact of the smoke-free policy upon ED visits by asthmatic youth are more robust. A study in Ireland evaluating the impact of the nationwide smoke-free policy showed a larger reduction in asthma-related ED visits (RR = 0.60) for the younger age group compared to overall asthma-related ED visits (RR = 0.85) [33]. This is consistent with our study findings of a larger reduction in asthma-related ED visits in children (RR = 0.75), compared to overall asthma-related ED visits (RR = 0.81). In another study in England smoke-free policies were temporally-related to a 12.3% reduction in childhood asthma-related ED visits [27]. A study in the US, which reviewed data among youth from the National Health and Nutrition Examination Survey (NHNES) between 1999 and 2006, observed a significant reduction (OR = 0.66) in asthmatic symptoms related to the implementation of smoke-free laws [34]. In Scotland, a trend for increasing childhood asthma-related ED visits (+5.2% per year) was observed prior to smoke-free law implementation, but a significant decrease in childhood asthma-related ED visits (-18.2% per year) was found after the law was implemented, yielding a net reduction of 13% per year following the smoke-free policy implementation [35]. Among children in our current study, we found that an upward trend in ED visit rates (+1.1% per month) for asthma prior to the passage of the smoke-free law was reversed, resulting in a downward trend (-0.4% per month) following the implementation of the law.

The findings that asthma-related ED visits were significantly reduced after the smoke-free law implementation, but the COPD-related visits were not significantly reduced, is not surprising. The lack of findings with COPD-related visits confirms findings from a recent meta-analysis [29]. Typically COPD is associated with irreversible or minimally reversible airway obstruction, unlike the bronchospasms in asthma, which can be severe but may be completely reversible. Exposure to triggers such as SHS may precipitate acute bronchospasms in asthmatics and is more likely to increase respiratory symptoms but not acute bronchospasms in people with COPD. Acute exacerbations of asthma often require emergency treatment or hospitalization to reverse. In communities with strong smoke-free laws, the changes in COPD-related hospital and ED admissions may be seen 12 months or more after implementation of the law [36]. This likely reflects the slower improvement seen in COPD symptoms and exacerbations as SHS smoke exposure declines. This may also explain the variable effects that are noted in studies of smoke-free laws on COPD adverse events. Investigators in Ireland, using time series analysis, evaluated the impact of the nationwide smoke-free law and found an effect only for COPD mortality only among females [37]. An ecological analysis of hospital discharge rates in Texas found a reduction for COPD hospital discharges only for white patients after implementation of the smoke-free law [38]. The greatest impact of smoke-free laws on COPD may be the effect they have on encouraging smokers to quit smoking, thus reducing the risk of COPD progression. This impact would be seen over the longer term and may be variable among the affected populations, consistent with the empirical data from population-based studies.

Our finding of decreased asthma-related ED visits following implementation of a statewide smoke-free law is biologically plausible and consistent with the known causal relationship between cigarette smoke exposure and exacerbation of pulmonary disease symptoms [39] as well as with previous observations of the relationship between smoke-free policies and reductions in SHS exposure and respiratory symptoms. The WHO estimated that smoke-free policies are associated with 40% (widespread) to 80%-90% (in high exposure setting) reductions in SHS exposure [40]. This report has been supported by a systematic review of over 50 studies addressing the role of the smoke-free policies on exposure to SHS [16]. In this systematic review, the studies addressing the role of SHS exposure consistently found a reduction of SHS in public places (workplace, restaurants, and bars) of about 72% within one year of a smoke-free law implementation [16]. This review also reported reductions in both reported respiratory and sensory irritation symptoms after smoke-free law implementation in 10 studies [16]. These sensory symptoms included wheezing or whistling in chest; shortness of breath; cough; phlegm; red, teary, or irritated eyes; runny nose or sneezing; and sore or scratchy throat. An additional study, not included in the prior referenced review, is a cross-sectional telephone survey of 382 nonsmokers in the workplace, which showed a positive dose–response relationship between exposure to SHS and increased reports of respiratory ailments [41]. Another study, which evaluated the impact of workplace and pub smoke-free laws in Ireland, collected air samples and conducted pulmonary function tests among 81 employees of 42 pubs before, and 1 year after, the law was implemented [42]. Using particulate matter, benzene concentration, expired air carbon monoxide, and salivary cotinine, the researchers found a 90% reduction to SHS exposure (i.e., 83% reduction in particulate matter 2.5 μm or smaller, an 80% reduction in benzene concentration, a 79% reduction in expired air carbon monoxide, and an 81% reduction in salivary cotinine), a significant improvement in respiratory function, and a decrease in other respiratory symptoms in nonsmokers after implementation of the law [42].

Our study has several limitations. First, we did not measure actual SHS exposure in different environments around the community. Second, our study data was limited only to ED visits which were for the most part, ambulatory and did not include hospital admissions for the local hospitals. Third, other tobacco control efforts were occurring during the study period, which could have contributed to the decrease in smoking prevalence and to reductions in SHS exposure. For example, from 1999 through 2010, the per capita cigarette sales in MN declined by 40% and smoke-free homes increased from 64.5% (1999) to 87.2% (2010) [43]. Other activities included a marketing campaign for the state tobacco quitline and local clinic tobacco treatment services and a 2004–2007 mass media campaign focused on the hazards of SHS. Despite these limitations, the data presented does parallel the 22% postban reduction of respiratory ED visits associated with the smoke-free law of other geographic locations [26, 30, 31].

## Conclusion

Following the enactment of a smoke-free workplace law we observed a reduction in asthma-related ED visits for both adults and children. This finding underscores the importance of comprehensive smoke-free laws and policies for improving public health and reducing health care costs. Communities need to continue to advocate for smoke-free laws and policies in order to reduce the burden of disease caused by SHS.

## Abbreviations

COPD: Chronic obstructive pulmonary disease
ED: Emergency department
MN: Minnesota
REP: Rochester Epidemiology Project
SHS: Secondhand smoke.
